# 1199. Effectiveness of Non-carbapenem vs Carbapenems Empiric Therapy for Extended-Spectrum β-Lactamase (ESBL)-producing Enterobacterales (PE) Infections in Non-ICU patients: A Real-world Investigation in a Hospital with High-prevalence of ESBL-PE

**DOI:** 10.1093/ofid/ofad500.1039

**Published:** 2023-11-27

**Authors:** Amy Y Kang, Mary W Elkomos, Danny Pham, Michelle Guerrero, Loren G Miller

**Affiliations:** Chapman University School of Pharmacy, Irvine, CA, Irvine, California; Chapman University School of Pharmacy, Laguna Hills, California; Huntington Hospital, Garden grove, California; Memorial Hermann - Texas Medical Center, Houston, Texas; Lundquist Institute at Harbor-UCLA Medical Center, Los Angeles, California

## Abstract

**Background:**

Surviving Sepsis guidelines recommend empiric broad-spectrum therapy that covers likely pathogens. However, among hospitalized, non-ICU patients with less severe clinical presentations, it is unclear whether effective empiric antibiotics lead to improved outcomes.

**Methods:**

We reviewed records of adult non-ICU patients admitted to a large urban medical center with confirmed ESBL-PE infection between Jan 2019 and Aug 2020. We excluded patients from our analysis if: antibiotics were started >48 h from the first positive culture; culture was polymicrobial; hospital length of stay was < 24 h; or positive cultures likely represented colonization. We grouped patients into receipt of empiric carbapenems or not. Our primary outcome was time until clinical stability from the first dose of the empiric antibiotic (in hours). Secondary outcomes included early clinical response and 30-day all-cause hospital readmission.

**Results:**

Among 448 patients with ESBL-PE isolates during our study period, 143 met study criteria. Of these 143, 60 (42%) received empiric carbapenem therapy. Median age was 59 y, and 67% were Hispanic. The most common source was urinary (71%). Carbapenem receipt was associated with previous antibiotics within 6 months of admission (55% vs 28%, p< 0.01) and history of ESBL (57% vs 17%, p< 0.01). Most common empiric therapy in the non-carbapenem group was ceftriaxone (59%). We found no difference in hours until clinical stability from the first dose of the empiric antibiotic between carbapenem and non-carbapenem groups (24 (IQR 8-40) vs 25 (6-75), p=0.77). We also found no difference in early clinical response between groups (88% vs 90%, p=0.78). We found a non-significant trend towards higher 30-day all-cause hospital readmission in the carbapenem group (17% vs 8%, p=0.13).

**Figure:**
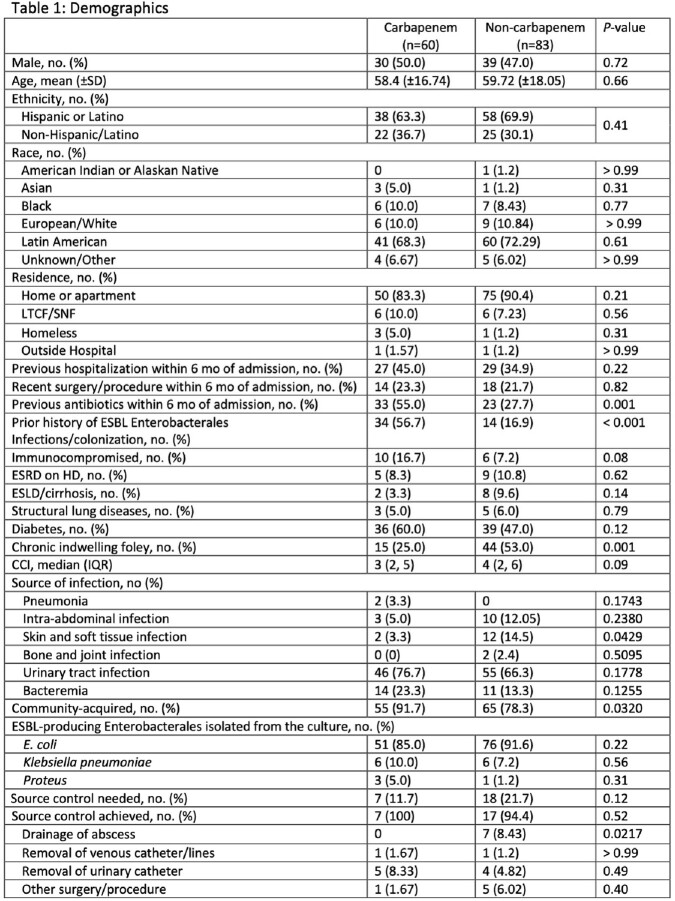

**Figure:**
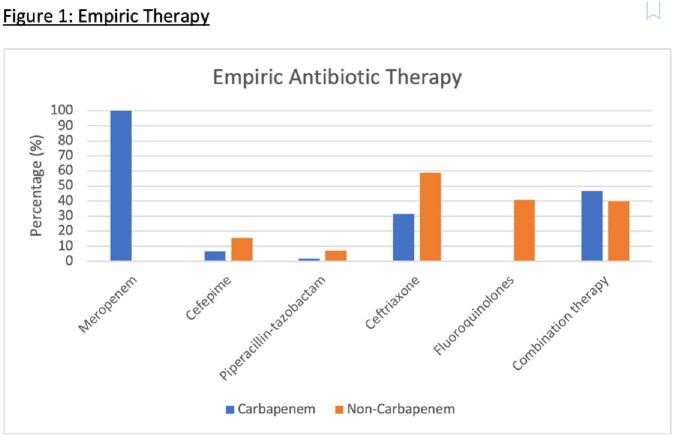

**Figure:**
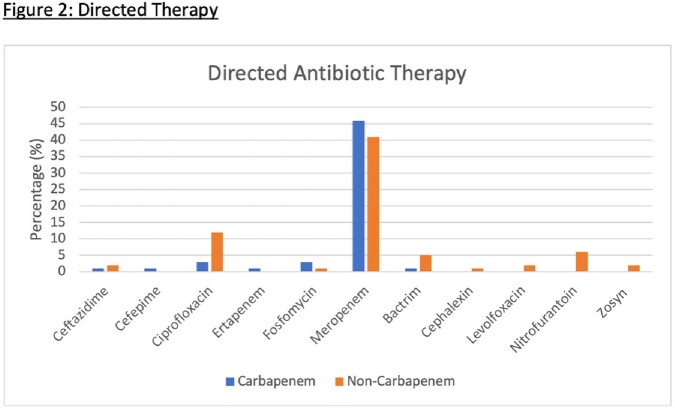

**Conclusion:**

Among hospitalized non-ICU patients with ESBL-PE infection, we found no difference in time to clinical stability after the first dose of the empiric antibiotic between those receiving carbapenems and those who did not. Our data suggest that empiric carbapenem use may not be an important driver of clinical response in patients with less severe ESBL-PE infection. Our findings may have important antibiotic stewardship implications.

**Disclosures:**

**Amy Y. Kang, Pharm.D., BCIDP**, Paratek: Grant/Research Support **Loren G. Miller, MD MPH**, ContraFect: Grant/Research Support|GSK: Grant/Research Support|Medline: Grant/Research Support|Merck: Grant/Research Support|Paratek: Grant/Research Support

